# The Costs of Exclusion: Recognizing a Role for Local Communities in Biodiversity Conservation

**DOI:** 10.1371/journal.pbio.0050289

**Published:** 2007-10-23

**Authors:** Marc Ancrenaz, Lisa Dabek, Susan O'Neil

## Abstract

Two cross-cultural, community-based conservation initiatives in Borneo and Papua New Guinea incorporate poverty eradication into their biodiversity conservation programs in areas harboring some of the world's most endangered species.

There is little debate that unchecked human population growth and the development of “modern” societies are responsible for the current biodiversity crisis. To stop the growing loss of biodiversity, global conservation efforts have mostly focused on creating protected areas free of human influence [1]. But many of these protected areas are also in crisis. In most cases, their long-term viability depends on the integrity of complex ecological processes that stretch well beyond their geographical boundaries. Efficient conservation initiatives need to be undertaken at the landscape level, incorporating multiple-use habitats where people and wildlife co-habit [2]. Since most traditional conservation efforts were typically designed to exclude human residents, they have often failed to actively involve groups of people living within or near protected areas. This failure to consider the interests of local communities has resulted in a general lack of support for conservation and subsequent degradation of protected areas [3]. Theories, rationales, and underlying principles about ways to integrate conservation and development are fueling passionate debate at many levels, but convincing documentation of successful implementation is still scarce [3–5]. Since substantial biodiversity is still occurring outside of protected areas, we believe that poverty eradication and biodiversity conservation are intimately interconnected. Addressing these two challenges simultaneously remains one of our best hopes for achieving tangible and durable results [6]. Here we describe two cross-cultural and inclusive community-based conservation programs in Borneo and Papua New Guinea (PNG) that were designed with these factors in mind.

## The Kinabatangan Orang-utan Conservation Project

**Figure 1:**
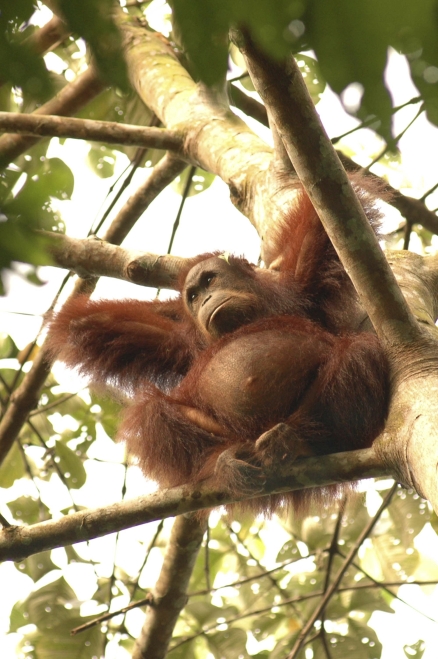
At eight years old, orang-utans like Etin, who lives in the KOCP intensive study site in the Lower Kinabatangan Sanctuary, start wandering alone in the forest to find a new territory. (Image: Jamil Sinyor)

The two extant orang-utan species, Pongo pygmaeus in Borneo (Figure 1) and Pongo abelii in Sumatra, are facing extinction due to the loss of the ecological integrity of the islands' lowland ecosystems [7]. The Malaysian state of Sabah (north Borneo), which is one of the species' major strongholds, harbors 11,000 orang-utans [8], about one-fifth of the Bornean population. About 60% of the animals are surviving outside of protected areas, in secondary forests that are exploited by indigenous communities and local industries, resulting in a direct conflict of interest between the needs for preserving this iconic species and the needs for human development.

In the Kinabatangan floodplain (east Sabah), past and recent exploitation of the natural resources (timber extraction, conversion to agriculture, etc.) have destroyed more than 80% of the original forest cover, degraded and fragmented natural ecosystems, caused environmental problems such as river pollution, depleted timber and wildlife resources, increased human-wildlife conflicts, and reduced the area available for the development of new economic activities. Yet the remaining forests still support a remarkably high abundance and diversity of wildlife, including ten primate species (including 1,100 orang-utans [9]), Bornean elephants, estuarine crocodiles, and more than 300 bird species. These species live in a mosaic of agriculture (mostly palm oil plantations), human-made habitat, and natural forests under different levels of degradation.

Until recently, the absence of in-depth field studies on the relationships between orang-utans and disturbed habitats impaired efforts to design and implement sound conservation strategies for this species in non-primary forests. In 1998, Hutan (a French nongovernmental organization [NGO]) and the Sabah Wildlife Department (SWD) initiated the Kinabatangan Orang-utan Conservation Project (KOCP) to rectify this situation. A small research center was established in the village of Sukau, and a permanent study site was set up in the forest. Today, KOCP employs 40 full-time research assistants, all from the local community.

At first, our research activities met resistance from most villagers. With their means of subsistence seriously degraded (by water pollution and the depletion of fisheries resources and forest products), most people viewed the proposed Lower Kinabatangan Wildlife Sanctuary as an attempt to lock up scarce resources that are essential for their own survival. And because crop-raiding elephants and orangutans upset the frail economy of many households, villagers viewed these species as pests, a sentiment that was further exacerbated when elephants devastated local graveyards by trampling down and pulling out tombs. Many Kinabatangan inhabitants asked, “Why give land to the orang-utans and elephants, and not to people?”

**Figure 2:**
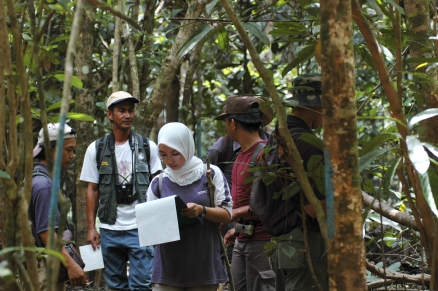
KOCP conducts orang-utan ecological fieldwork during a joint program with staff from Sabah parks in the Lower Kinabatangan Wildlife Sanctuary. (Image: Jamil Sinyor)

Since orang-utans cannot survive outside of natural forest, it became clear that preserving this species in Kinabatangan would require the development of ecosystem management programs that embraced a wider perspective than the species itself and considered the needs and aspirations of the local communities. A prerequisite for local support of wildlife preservation is the recognition of the intrinsic value and uniqueness of species that inhabit the area. To build support for the project, the KOCP local research assistants organized in-depth consultations with community members to identify major challenges as well as the threats posed by local wildlife. With villagers' input, the KOCP started to implement an integrated and multidisciplinary strategy, combining scientific research, community engagement, capacity building, education, and policy formulation. This process involves in-depth training sessions in field research (Figure 2), community participation techniques, sustainable development, environmental education, computer skills, English language skills, and project management. These efforts have produced an effective network of Sabahan partners in government agencies, NGOs, and research institutions, as well as involving private stakeholders.

Since the project started, we have witnessed a gradual decline of illegal and nonsustainable use of the remaining natural resources of Kinabatangan by indigenous people. Most encroachments today originate from outside the local communities (private industry and nonresident people). More importantly, community members gradually started to realize the importance of preserving the last forests for their own well-being and to support the creation of the Lower Kinabatangan Wildlife Sanctuary through local media and other means. In 2005, 26,000 ha of forests were eventually protected and placed under the jurisdiction of the SWD. We attribute this positive change in attitude to education campaigns raising awareness about the importance and uniqueness of wildlife species found in the area (orang-utans being the major icon), and in large part to encouraging local community members' active participation in conservation efforts and strategy.

For example, 20 KOCP research assistants were officially recognized as honorary wildlife wardens to help the SWD enforce the new Sanctuary Management Plan. Wardens patrol the area to control human encroachments within and beyond the sanctuary, monitor wildlife, and mitigate conflicts, and they hold events to raise conservation awareness in schools and the community. This alliance between a state agency and community members lays the foundation for securing the long-term management of the sanctuary. Financial losses resulting from wildlife crop-raiding activities were a major impediment to building local support for wildlife conservation. In 2000, a team of seven KOCP research assistants created the Elephant Conservation Unit to alleviate wildlife conflicts and to increase tolerance for wildlife-induced damages. This community-based squad implemented nonlethal control strategies consisting of both active and defensive mitigation measures (see [10]), trained local farmers in mitigation techniques, and allocated micro-loans to small-scale landowners to build fences. The unit also investigates the ultimate causes for raiding activities through scientific studies of elephant ecology—including feeding strategies, home range patterns, and identification of bottlenecks—to prevent future raids. These control activities engendered a better acceptance of the animals—leading to a complete halt of elephant shooting as a means of crop protection and an 80% reduction of economic losses due to crop-raiding activities in the area of Sukau over five years.

Nature-based tourism provides another opportunity to give the local community a stake in conservation. However, in most cases, “ecotourism” ventures are commercially run by private operators and result in indirect and sometimes negligible benefits to local communities [11]. In 2001, the SWD, Hutan, and the community of Sukau, with the initial funding from the Danish Cooperation Agency, launched Red Ape Encounters (RAE), a community-based ecotourism model for integrating wildlife preservation with local economic development through orang-utan viewing. The SWD awarded the community the exclusive right to use a part of the sanctuary to develop ecotourism activities, and a for-profit company owned by the people of Sukau was registered in 2005. A transparent benefit-sharing mechanism ensures that the revenues generated by RAE ecotourism activities profit all its members, not just RAE's personnel. Further integrating the ecotourism project into the local economy, RAE contributes 4% of its tourism revenue to two funds (the Community Tourism Development Fund and the Community Conservation Fund), each managed by a different village committee [12].

Conservation initiatives in Kinabatangan currently focus on implementing a general bio-monitoring program in order to assess the general health of the ecosystem (forest coverage and regeneration), as well as orang-utan and other wildlife population trends. Our current results show that orang-utans can survive in the degraded forests of Kinabatangan, that the elephant population has almost doubled over a ten-year period, and that forest loss due to human encroachments is decreasing. Although it is still too early to quantify precisely how the ecosystem benefits from all these efforts, the partnerships developed between village members, government agencies, and other players active in Kinabatangan provides the best possible long-term sustainable model that simultaneously considers the needs of the communities and those of wildlife.

## The Tree Kangaroo Conservation Program

On the Huon Peninsula of PNG, the conservation issues are very different, but the strategy is the same—community involvement. With more intact forest than other tropical countries, PNG is considered by Conservation International to be one of three remaining tropical wilderness areas [13]. The Huon Peninsula is a remote and incredibly diverse region of PNG with a steep elevation gradient from coral reefs to cloud forests—a rare habitat worldwide with over 60% of the total area in Asia Pacific and a significant portion of that in PNG [14]. Many forested regions of PNG have been exploited by outside private industries for timber and mining resources. On the Huon Peninsula, however, the rugged terrain has discouraged road building, and thus encroachment by outside industries has been minimal, although the potential for commercial extraction activities is increasing. Future threats include large-scale logging and mineral extraction as world demand increases and new areas are targeted [15]. Current threats to the Huon Peninsula are more localized, and include increasing village populations, subsistence hunting, and small-scale subsistence logging and resource extraction.

When the Tree Kangaroo Conservation Program (TKCP) started working on the Huon Peninsula in 1996, we found that although the endemic Matschie's tree kangaroo (Dendrolagus matschiei) was endangered [16], there was still time to prevent commercial over-exploitation of resources, with locally focused solutions. Conservation depended on local education and outreach to raise awareness about the threats to tree kangaroo survival and to identify ways the local community could help sustain the populations. The indigenous people have a stake in maintaining healthy tree kangaroo populations, because the animals are part of the local diet and their fur is used for ceremonial dress. TKCP's priority on the peninsula is to create a locally managed conservation area to prevent habitat destruction and wildlife species decline.

**Figure 3:**
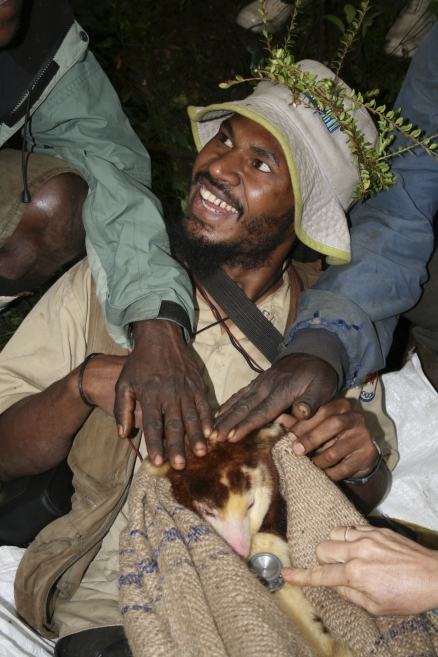
Gabriel Porolak of PNG prepares to outfit a tree kangaroo with a radio collar. (Image: TKCP)

The endangered Matschie's tree kangaroo, which is endemic to the cloud forest on the Huon Peninsula, is the flagship species for this community-based conservation program. Other threatened Huon Peninsula species include the endangered long-beaked echidna (Zaglossus bruijni), the New Guinea harpy eagle (Harpyopsis novaeguineae), the vulturine parrot (Psittrichas fulgidus), and the endemic bird of paradise species Huon astrapia (Astrapia rothschildi) [17]. In 2001 and 2003, biodiversity inventories were conducted at different elevations within the proposed conservation area to provide baseline data for monitoring species presence. Tree kangaroo population densities have been surveyed at three sites using distance sampling techniques. TKCP will also be implementing a series of biodiversity and cultural metrics to monitor success of the conservation area and provide measures to monitor tree kangaroo populations (Figure 3) and to evaluate biodiversity benefits. Surveys of local project assistants completed before and after the biodiversity studies indicated that involvement in these conservation projects changed their perception of the intrinsic value of the habitat and species unique to the area and increased their commitment to conserving the wildlife (unpublished data).

With over 95% of the land in PNG owned by the indigenous people [18], conservation programs must win the support and involvement of local people to succeed. Ecotourism is not a viable option in these communities, due to their remoteness and lack of transportation infrastructure, which also limits access to government services. TKCP has worked with the landowners of the YUS (Yupno, Uruwa, and Som rivers) local-level government for over ten years, aided by staff recruited from local university graduates and the YUS community. Through these alliances, TKCP explored social or human service benefits that would work within the existing landscape and culture and decided to focus on improving local education and healthcare.

TKCP provides both immediate resources and long-term investment in education within the communities, mostly funded by outside grants to TKCP. The project sponsors local students at a teacher training college with the agreement that after graduating, they will teach in YUS village schools for at least six years. Sponsoring local students to obtain the necessary education to return to their community not only addresses the difficulty of recruiting educated outsiders to this remote area, but also builds the educational foundation of the local community. With more teachers in the community, at least three new schools have been able to reopen. Also, the local-level government and the YUS Education Committee have agreed to financially invest in the program to help even more students receive training each year and to alleviate the local teacher shortage.

These staff members then help plan and implement an annual teacher training workshop in the villages sponsored by TKCP. YUS teachers define the annual themes with TKCP staff and receive training in teaching methodology, environmental education, and conservation curriculum. Similar to KOCP, capacity building and training are integrated into all TKCP activities with staff and community members, including university programs, training workshops, conferences, and exchange visits with other communities and conservation programs.

Following the success of the education projects, TKCP visited villages and worked with the provincial healthcare workers in 2005 to identify outstanding health care needs. Many villages have no access to outside doctors, forcing the community to deal with most health care issues. TKCP is sponsoring workshops with the Provincial Health Department to train midwives and community heath workers through an initiative called “Healthy Village, Healthy Forest,” which acknowledges that conservation programs must address the health of the human community so the community can address the health of the environment.

In exchange for TKCP's support, local land owners have pledged portions of intact habitat on their land to establish the country's first conservation area, which now covers over 60,000 ha from sea level to 4000 m, which are off limits to hunting, resource extraction, and forest conversion of any sort (no large- or small-scale logging). Landowners have primarily pledged intact habitat at higher elevations or greater distances from a village, setting aside other areas of their forest for subsistence hunting. Landowners explain that this strategy creates “wildlife banks”—areas safe for wildlife to reproduce—that generate “interest”: offspring dispersing into hunting areas. This strategy is consistent with historical local cultural practices of leaving certain forest areas untouched and treated as “taboo”—a traditional conservation approach that had previously been curtailed by missionaries. Land near the villages continues to be used for subsistence farming and resource extraction. As a result of these pledged areas, TKCP staff and YUS landowners have reported an increase in tree kangaroo evidence (based on sightings, scat, and scratch marks on trees) and the return of wildlife species not seen on their land for generations (unpublished data).

The Conservation Areas Act of PNG, originally passed in 1978, provides the legal foundation for protecting sensitive species and habitats. Once landowners pledged their land to conservation, TKCP staff began working with the local, district, provincial, and national government, along with other NGOs and the PNG Department of Environment and Conservation, to formalize the YUS conservation area. Following approval of the conservation area by the national government, the management plans for the conservation area must be finalized. To achieve long-term species and habitat protection, local landowners have been involved in all aspects of developing the management plans including the mapping (geographic information system boundary mapping and locations of culturally and naturally significant areas), management rules, and fines. To ensure the community can successfully manage the conservation area over the long term, TKCP is helping YUS establish a community-based organization, which will maintain the link between conservation, education, and healthcare services. Acting as the liaison between the YUS communities and various government agencies, TKCP has the resources to implement the education, healthcare, and conservation projects that are driven by local decision making but hampered by lack of access to government bodies and urban centers.

## Lessons Learned

KOCP and TKCP have different conservation issues in very different landscapes, yet both embrace a similar process and philosophy of involving local communities in directing their missions. TKCP is working in a relatively intact area conserving species and forests, which would otherwise be affected by gardening and resource use, while working to avoid future conflicts between the communities and outside pressures. Meanwhile, KOCP is working in a highly fragmented landscape with current human-animal conflicts and with intense human pressure placed on the last natural resources of the area. Meeting human needs and respecting biodiversity in both areas is the means to a conservation end.

The Kinabatangan experience shows that in the absence of hunting (traditionally, local communities do not hunt wildlife for pet trade or for food—except deer), a wide array of wildlife (including orang-utans) can survive in relatively small patches of degraded and fragmented multiple-use forests. Hunting remains the major threat to wildlife in tropical forests worldwide where this activity goes uncontrolled. Since orang-utans depend directly on natural forests, their long-term survival depends on protecting the various forest remnants from further destruction. Financial incentives brought by conservation and ecotourism activities are important factors explaining the increasing support for orang-utan conservation in Sabah. However, making the orangutan a symbol of the State's natural heritage was even more effective in attracting interest from various stakeholders (local communities, private industries, and land deciders alike) and in raising awareness about the species' fate. Eventually building a trustful collaboration with government agencies and empowering selected community members in the management of their natural resources appears to be the most promising approach to securing the future of Kinabatangan. The on-going monitoring will provide the necessary data to document the measurable impacts of all these efforts on the general ecosystems and biodiversity.

For the Huon Peninsula, the primary benefit for the local community is that TKCP can serve as a link between their remote region and the different levels of government in PNG. TKCP is helping the YUS community receive desperately needed and deserved human services in exchange for managing their forests in a sustainable manner. Because the YUS conservation area will be the first of its kind in PNG, the YUS community is now also seen as a leader in the country and is receiving unprecedented attention nationally and internationally.

For community-based conservation approaches to succeed, they must make a long-term commitment, allow for flexibility in responding to new situations and opportunities, and, more importantly, establish a strong physical presence on the ground. Sharing and experiencing the daily conditions of community life, learning the vernacular language, respecting the local traditions, understanding current and historic use of the forests, and—above all—valuing the dignity, knowledge, and connection of the people to their land and their survival are essential tools to forge alliances and develop trust with local people and, more generally, to eventually demonstrate the value of conserving wildlife and its habitat. Last but not least, we need to recognize that a strategy that is successful in a given scenario at a given time is not necessarily replicable to other situations. Although it is crucial to draw conclusions from the different grassroots conservation initiatives existing today, it is unlikely that a simple and unique path exists for reconciling human development and biodiversity conservation.

## Abbreviations

KOCP: Kinabatangan Orang-utan Conservation Project
NGO: nongovernmental organization
PNG: Papua New Guinea
RAE: Red Ape Encounters
SWD: Sabah Wildlife Department
TKCP: Tree Kangaroo Conservation Program
YUS: Yupno, Uruwa, and Som local-level government region.
